# Splenic infarction in sickle cell trait: A comprehensive systematic review of case studies

**DOI:** 10.1002/jha2.248

**Published:** 2021-07-11

**Authors:** Jamal M. Jefferson, Wynton M. Sims, Nkeiruka Umeh, Yen Ji Julia Byeon, Khadijah E. Abdallah, Vence L. Bonham, Rakhi P. Naik, Kim Smith-Whitley

**Affiliations:** 1 National Human Genome Research Institute, Division of Intramural Research, Social and Behavioral Research Branch, National Institutes of Health, Bethesda, Maryland; 2 Division of Hematology, Department of Medicine, Johns Hopkins University, Baltimore, Maryland; 3 Division of Hematology and Director of the Comprehensive Sickle Cell Center, Children’s Hospital of Philadelphia, Philadelphia, Pennsylvania

**Keywords:** acute disease, asymptomatic condition, sickle cell trait, splenic infarction

## Abstract

Sickle cell trait (SCT), a commonly asymptomatic condition, has many associated clinical complications that upon presentation, can be very difficult to attribute to SCT. The effects of SCT on the spleen, for example, are not completely understood, though there have been a number of case reports detailing related complications in diverse populations. Our objective was to perform the first comprehensive case report review of splenic infarction in SCT patients to highlight the relevance of this seemingly rare condition. We conducted an extensive literature search reviewing case reports and case series of acute splenic infarctions from 1970 to 2020. This comprehensive search resulted in 54 articles with a total of 85 individuals. The ages ranged from 7 to 65, 12% were female. Individuals were of African-American (26%), European (16%), South Asian (13%), Middle Eastern (7%), Latin American (7%), North or East African (4%), Mediterranean (4%), West African (1%), and unknown (22%) origins. Although splenic infarct in SCT patients has been associated with high altitudes, 39% of cases reporting altitude occurred below 3000 m. Among cases where HbS values were recorded, 88% occurred in individuals with HbS levels higher than 35%, suggesting that high HbS values may be a risk factor for splenic infarction. Our findings indicate that splenic infarct occurs across a wide range of demographic populations and environmental settings. While our understanding of SCT evolves, the findings here suggest that future advances in research and healthcare could benefit more from real-time surveillance and registry initiation for various SCT outcomes such as splenic infarct.

## 1 | INTRODUCTION

Sickle cell trait is a heterozygous state that results from the inheritance of one variant gene for sickle hemoglobin and a normal gene for adult hemoglobin. SCT is estimated to affect one to three million individuals in the United States and over 300 million individuals worldwide.[1] The global distribution of SCT, which varies widely by geographic region, is hypothesized to have been driven by the protection that SCT confers against falciparum malaria in malaria-endemic regions such as sub-Saharan Africa, India, southern Europe, and the Middle East.[2–4] In the United States, individuals who may have had ancestry in these regions, such as African Americans and Hispanic or Latinx/a/os, are more likely to be affected by SCT.[5]

Unlike as in sickle cell disease (SCD), the erythrocyte sickling does not generally occur in SCT carriers, and the carrier status has historically been described as benign. However, several high-profile cases involving SCT-associated clinical complications among athletes and military personnel continue to raise questions about the benignity of the heterozygous state. Research has suggested that some individuals living with SCT are at higher risk of certain conditions, including venous thromboembolism, chronic renal diseases, renal medullary cancer, hematuria, renal papillary necrosis, hyposthenuria, and splenic infarction.[6–8]

This review focuses specifically on SCT and splenic infarction, one of the most widely reported but possibly least understood complications associated with SCT [9]. The underlying pathophysiology is thought to result from subacute erythrocyte sickling in the spleen in settings of low oxygen tension [10]. The aim of this study is to conduct a case study literature review of splenic infarction in individuals with SCT and comprehensively examine the risk factors for the development of this complication in children and adults with SCT.

## 2 | METHODS

**FIGURE 1:**
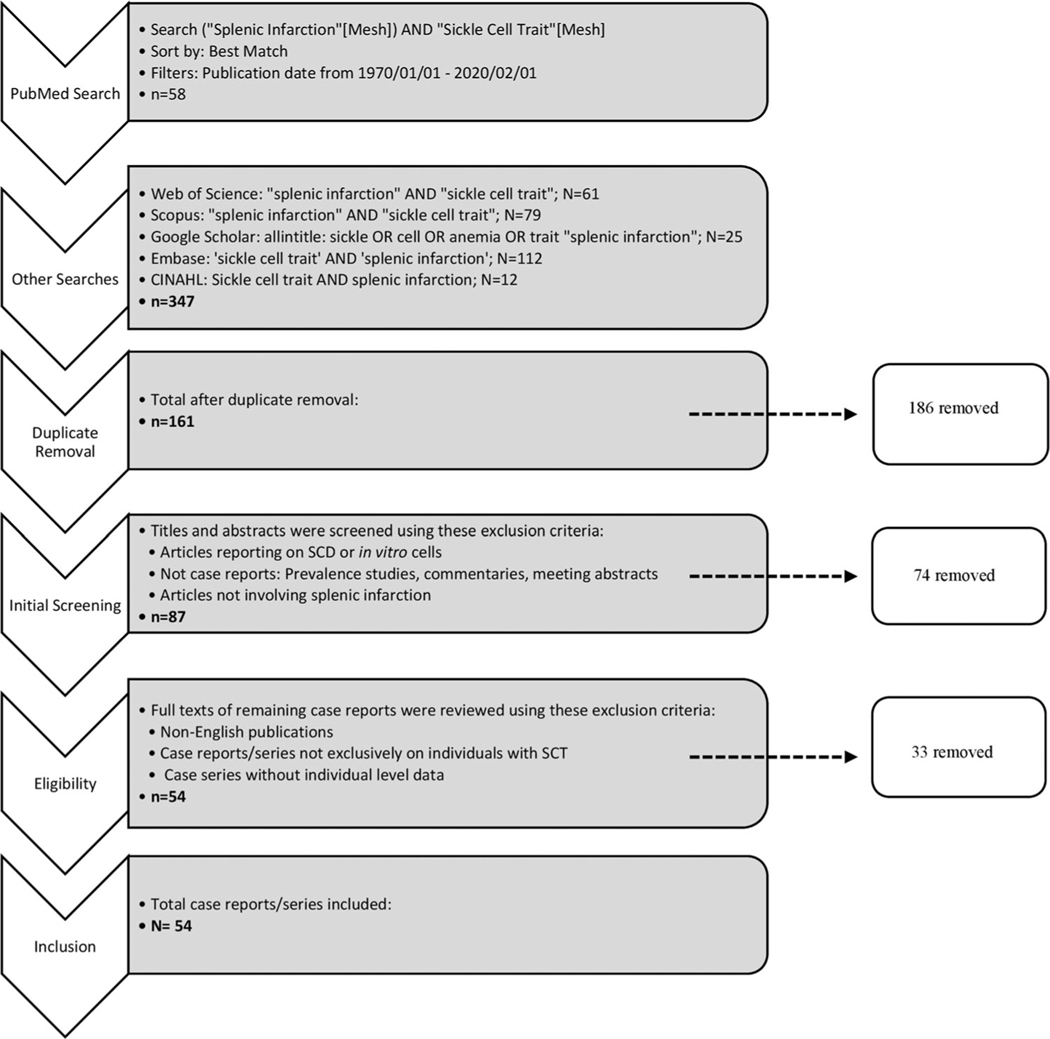
Method of systematic review

A comprehensive literature review of peer-reviewed journal articles published between January 1, 1970 and February 1, 2020 was conducted (Figure 1). The literature search was conducted using bibliographic databases, including PubMed, Web of Science, Scopus, Google Scholar, Embase, and CINAHL. The following search terms were used: (“Splenic Infarction”[Mesh]) AND “Sickle Cell Trait”[Mesh]; “splenic infarction” AND “sickle cell trait”; “splenic infarction” AND “sickle cell trait”; sickle OR cell OR anemia OR trait “splenic infarction”; “sickle cell trait” AND “splenic infarction”; “sickle cell trait” AND “splenic infarction,” respectively.

Five reviewers (JMJ, WS, and NU, KA, YJJB) screened articles based on predetermined criteria. Duplicate articles and publications not related to splenic infarction were excluded. Titles and abstracts were then assessed for eligibility to be included in the literature review. The following were excluded: (1) research articles that reported exclusively on patients with SCD or in vitro cells; (2) articles that were not case reports or case series, including meeting abstracts, prevalence studies and commentaries; and (3) case reports or case series not involving splenic infarction. The full texts of all remaining case reports and series were examined. After a systematic and comprehensive review of these full texts, case reports and series were excluded on the following parameters: (1) non-English publications; (2) case reports and series not exclusively on individuals with SCT (e.g., related to spherocytosis), and (3) case series without individual-level data.

## 3 | RESULTS

The 1970–2020 literature searches and reference mining yielded 347 publications from PubMed (*n* = 58), EMBASE (*n* = 112), Scopus (*n* = 79), Web of Science (*n* = 61), CINAHL (*n* = 12), and Google Scholar (*n* = 25). After removing duplicates, we retrieved 161 articles. Each reviewer conducted two separate rounds of exclusions—the first round excluding publications based on titles and abstracts and the second excluding based on a full text review. From these exclusions, 54 articles (11 case series and 43 case reports) were identified and abstracted (Figure 1). No case-control, cross-sectional, prospective cohort, or longitudinal study of sickle cell trait-related splenic infarction were found.

From these 54 articles, we abstracted 85 cases of splenic infarction in individuals with SCT. Of the 85 individuals, 75 (88%) were male and 10 (12%) were female. A broad range of ages (7–65 years old) was represented. Thirteen (15%) individuals were 18 years or younger, 22 (26%) were between 19 and 25 years old, 24 (28%) were between 26 and 35 years old, 16 (19%) were between 36 and 45 years old, and 9 (11%) were 46 and older, and one individual’s age was unknown (1%).

Ethnicity data were examined for 85 subjects [9, 11–54]. We divided individuals into population groups based on geographic areas of descent for purposes of analysis, acknowledging that population categorization can be an arbitrary process that may yield varying results depending on context. Twenty-two (26%) were of African descent, 14 (16%) were of European descent, 11 (13%) were of South Asian descent, 6 (7%) were of Middle Eastern descent, 6 (7%) were of Latin American descent, 3 (4%) were of North or East African descent, 3 (4%) were of Mediterranean descent, 1 (1%) was of West African descent, and 19 (22%) were of unknown descent. The demographics of all individuals included in the review are summarized in Table 1.

As outlined in Tables 2 and 3, the geographic location for the onset of the splenic infarction was examined for 85 cases [9, 11–23, 25–41, 43–52, 54–61]. Thirty-two (38%) cases occurred in the United States and 41 (48%) occurred internationally (Table 2). Twelve of the cases reported internationally occurred in Japan (on or near Mt. Fuji), while the remaining cases occurred in India, Iran, Italy, Ethiopia, Saudi Arabia, the Canary Islands, Spain, Peru, the Himalayas, Sri Lanka, Greece, Canada, and Ecuador. One splenic infarction case transpired in transit while the individual was on a pressurized airplane traveling from California to New Jersey [34] (Table 3).

Altitude levels were reported for 59 individuals [9, 11, 13, 17–23, 26–28, 30, 34, 37, 39, 41, 43, 44, 46–48, 50–52, 55, 57, 59–61]. Of the 59 cases reporting altitude, 2 (3%) cases occurred under 1000 m, 4 (7%) cases occurred between 1001 and 2000 m, 17 (29%) cases occurred between 2001 and 3000 m, 27 (46%) cases occurred between 3001 and 4000 m, 2 (3%) cases occurred above 4000 m, and 7 (12%) cases contained a range or ambiguous altitude levels. In evaluating exercise alone, 29 (34%) of the individuals were physically active during their infarction, 43 (51%) were not physically active, and 13 (15%) cases were unknown. Additionally, most of the cases involving physical activity occurred at high altitudes. Out of those 29 individuals who were physically active and experienced a splenic infarction, 16 (55%) occurred at an altitude > 3000 m, 9 (31% %) occurred at an altitude < 3000 m, 1 (3%) occurred at 3000 m and 3 (10%) occurred at an unknown elevation.

Hemoglobin S (HbS) levels were reported for 48 cases [9, 11, 12, 15–17, 19–27, 29, 30, 32, 33, 35, 36, 38–41, 43, 46–51, 53, 54, 56–58, 62, 63]. The percentage of HbS ranged from 29.8% to 46.5%. The following HbS percentages occurred in 48 cases: HbS below 35% (4 cases or 5%), HbS between 35% and 39.9% (16 cases or 19%), HbS between 40% and 45% (26 cases or 31%), HbS greater than 45% (2 cases or 2%), and HbS unknown (27 cases or 43%). All the descriptive characteristics of splenic infarction are summarized in Table 2, while table 3 presents the individual case reports.

Although symptoms varied in each case, most individuals presented with more than one symptom and/or sign of splenic infarction, including abdominal or left upper quadrant abdominal pain (74% or 95%), vomiting (30% or 38%), respiratory issues (e.g., shortness of breath, pain) (19% or 24%), nausea (16% or 21%), left flank pain (5% or 6%), and jaundice (2% or 3%). Individuals with past medical histories available had reports of alcoholism, gout, obesity, glucose-6-phosphate dehydrogenase (G6PD) deficiency, cocaine and heroin use, chronic pancreatitis, and sleep apnea.

## 4 | DISCUSSION

Although SCT is largely considered a benign carrier state, reports of clinical complications in rare circumstances exist [8]. More frequently, chronic complications of SCT such as chronic kidney disease and venous thromboembolism are reported in large epidemiologic studies [33, 64, 65]. However, acute complications of SCT, such as splenic infarction, are considerably rarer, and thus the literature is limited to case reports and series. We present the first comprehensive case study review of splenic infarction in SCT and find that the demographics and clinical presentations of this complication in individuals with SCT have considerable heterogeneity.

High altitude environments with low oxygen tension are recognized as a potential factor in the development of splenic infarction in people with SCT, such as during mountain climbing or travel in unpressurized airplanes [66]. In our study, 49% of cases reporting altitudes occurred at greater than 3000 m, and a number of these cases demonstrated resolution of symptoms upon descent to a lower altitude. However, 39% of cases reporting altitudes occurred below 3000 m, suggesting that altitude is not the sole environmental risk factor for this complication [9, 11, 13, 14, 22, 26, 30, 41, 44, 50, 51, 60, 61]. It is therefore difficult to deduce an approximate altitude at which splenic infarction is likely to occur and is important to acknowledge the possibility of infarction in the absence of a high altitude or hypoxic environment.

There is controversy over whether those of non-African ancestry with SCT are more susceptible to splenic infarcts. Several prior reports have suggested that splenic infarction is more likely to occur in SCT individuals of European descent compared to those of African descent [18, 38, 66]. Genetic differences, such as frequency of *α*-thalassemia mutations, have been postulated to underlie this difference [47]. In our current review, though we found that 22/83 cases were African-American, this area is limited by the small number of studies and ambiguous definitions of population categorizations across studies. In our current review, we found that there may be an under-reporting in AAs overall [9, 15, 16, 18, 22, 28, 31, 34–38, 44, 45, 53]. Underreporting may be due to misdiagnosis as presenting symptoms that are similar to “mountain sickness.” Nonetheless, our study demonstrates that SCT-related splenic infarction appears across multiple geographic-descent populations (Table 3).

As with ancestry, the association between sex, as a predisposing factor, and splenic infarction is unclear. Our review of the literature confirms that both men and women are at risk of splenic infarction. This contrasts with what was observed in Goodman et al. in which all the patients were male [66]. In our review, there were ten reported cases occurring in women, three of which occurred in high altitude environments (> 3000 m) [22, 34, 37]. Although there is more frequent reporting of males, the potential reasons underlying this phenomenon are manifold; for example, one cause among many may be related to more men than women historically engaging in mountain climbing and other strenuous activities at low oxygen levels [21].

The amount of circulating HbS may influence the prevalence of clinical complications in SCT. Co-inheritance of alpha-thalassemia, which lowers HbS levels, has been found to decrease urinary concentrating dysfunction among individuals with SCT [47]. In SCT carriers without alpha-thalassemia, the average HbS percent is between 35% and 45%. In our review, we found that most of the reports with recorded HbS values (44/48 or 88%) occurred in individuals with HbS levels above 35%; therefore, high HbS values may predispose individuals to splenic infarction in SCT. Additionally, several case reports noted other potential risk factors such as drug use, sleep apnea, and infection, which may contribute to the pathophysiology of splenic infarction in SCT individuals [14, 18, 38, 56].

The pathophysiology of splenic infarction in SCT is not clear. The risk of pulmonary embolism has been noted to be higher in individuals with SCT compared to those without [8], and a few of the case reports in our review did mention a history of pulmonary embolism or infarction in SCT carriers who also experienced splenic infarction [24, 39, 49]. While chronic hypercoagulability likely plays a role in venous thromboembolism, it is not known whether acute arterial complications such as splenic infarction also have a common underlying mechanism. Given the limitations of case series and reports, no definitive conclusions about clinical risk factors for SCT-related splenic infarction can be made. In general, splenic infarction in individuals with SCT is a rare event, no comprehensive research studies have been conducted of this clinical outcome, and our review was limited to case reports and case series.

There is a need for a more comprehensive reporting of splenic infarction and specifically, a better understanding of presenting symptoms and physical examination findings to reduce its misdiagnosis (e.g., mountain sickness) and improve clinical outcomes. We have not discussed clinical presentation of the cases. Future studies and more data collection, possibly through the initiation of patient registries, are needed to better characterize risk factors for this complication in people with SCT and to determine optimal clinical management.

## Figures and Tables

**TABLE 1:** Demographics of SCT individuals who had a splenic infarction

Variable	Category	*N* (85)	%
Sex	Male	75	88
	Female	10	12
Age (years)	≤18	13	15
	19–25	22	26
	26–35	24	28
	36–45	16	19
	46+ Unknown	91	111
Population groups	African-American	22	26
	European	14	16
	South Asian	11	13
	Middle Eastern	6	7
	Latin American	6	7
	North & East African	3	4
	Mediterranean	3	4
	West African	1	1
	Unknown	19	22

**TABLE 2:** Descriptive characteristics of splenic infarction in 85 cases

Variable	Category	*N*	%
Onset location	US	32	38
	Internationally	41	48
	Not reported	12	14
Altitude level (m)	≤1000	2	2
	1001–2000	4	5
	2001–3000	17	20
	3001–4000	27	32
	>4000	2	2
	Ambiguous	7	14
	Not reported	26	31
HbS levels (%)	<35	4	5
	35–39.9	16	19
	40–45	26	31
	>45	2	2
	Not reported	37	44
Physical activity	Yes	29	34
	No	43	51
	Not reported	13	15
Aviation	Yes	19	22
	No	57	67
	Not reported	9	11
Splenectomy	Yes	25	29
	No	57	67
	Not reported	3	4

**TABLE 3:** Case reports of splenic infarction

Reference	Year published	Geographic location	Sample (age, sex, reported race/ancestral group)	Time of onset	Physical activity/aviation	Altitude level	Intervention/outcome/(additional comments)	Hb levels
O’Brien et al. [40]	1972	Mt. Washington, NH, USA	26, M, Sicilian (white American)	3 days after ascent	Physical activity: yes Aviation: no	760 m	*Splenectomy*: no Intervention unreported (patient was obese)	HbS: 42.4% HbF: 5%
King et al. [28]	1977	Los Angeles, CA, USA	58, M, Mexican	Unknown	Physical activity: no Aviation: no	No information	*Splenectomy*: yes Stayed in hospital 1 month (Patient was moderately obese, had a 15-year history of gout and had a transvenous demand pacemaker for bradycardia)	HbS: 31% HbA: 61%
Diep et al. [20]	1979	Colorado, USA	23, M, German/English ancestry (white)	30 minutes into arrival of Leadville, CO	Physical activity: no Aviation: no	3291 m	*Splenectomy*: yes Rapid recovery and home 1 week later	HbS: 39.7% HbA: 55% HbF: 2% HbF: 2%
Magnuson et al. [30]	1980	Minneapolis, MN, USA	37, M, African American	Unknown	Physical activity: no Aviation: no	Low (not specified)	*Splenectomy*: no Uneventful recovery; remained asymptomatic until one year later with onset of left-sided chest pain	Unknown
Buch et al. [15]	1982	Queens, NY, USA	32, F, African American	Spontaneous	Physical activity: no Aviation: no	Low (not specified)	*Splenectomy*: yes Uneventful recovery (patient hadiron deficiency anemia)	HbS: 32% HbA: 64.6% HbF: 1.5% HbA2: 1.9%
Callis et al. [16]	1982	Canary Islands, Las Palmas	13, M, Spanish	Upon ascent on cable car (within 8 minutes of ascent)	Physical activity: no Aviation: no	3555 m	*Splenectomy*: no Conservative therapy (misdiagnosed with mountain sickness but actually splenic infarction)	HbF: 3.8% HbA2: 2%
Cox [19]	1982	Pike’s Peak, CO, USA	20, M, white American	During descent by train	Physical activity: no Aviation: no	4297 m	*Splenectomy*: no Treated with nasogastric suction, IV hydration, and meperidine for analgesia; discharged on the 8th day	HbS: 41.2% HbA: 55.2% HbA2: 3.6%
Nussbaum et al. [38]	1984	Quito, Ecuador	36, M, White (Ecuadorian-born)	After ascent	Physical activity: no Aviation: no	3000 m and 5000 m	*Splenectomy*: no Conservative therapy used on multiple occasions (patient had a life-long history of exertional intolerance, chronic hemolytic anemia and pulmonary infarctions. He also noted a long history of ascending to high altitudes and experiencing jaundice; eventually moved to sea level and had no recurrence of symptoms)	HbS: 41.3% HbA: 55.6% HbF: 0.6% HbA2: 2.6%
Lane et al. [29]	1985	Colorado, USA Colorado, USA Colorado, USA Colorado, USA Colorado, USA Colorado, USA	18, M, Dutch-Sicilian descent (White) 18, M, Belgian-Spanish-Italian descent (White) 33, M, North European 37, M, Arab 19, M, Colombian 30, M, African American	<24 hours after arrival to Colorado 4 hours after arrival to Colorado < 12 hours after arrival to Colorado 8 hours after arrival to Colorado < 24 hours after arrival to Colorado 48 hours after arrival to Colorado	Physical activity: no Aviation: yes Physical activity: no Aviation: yes Unknown Unknown Unknown Unknown	1646 m 2134 m 3353m 3353m 1829m 3353m	*Splenectomy*: no *Splenectomy*: no *Splenectomy*: no *Splenectomy*: no *Splenectomy*: no *Splenectomy*: no	HbS: 40.5% HbA: 54.1% HbF: 1.2% HbA2: 4.2% HbS: 39.1% HbA: 55.3% HbF: 2.3% HbA2: 3.3% HbS: 41.3% HbA: 57.3% HbA2: 1.3% HbS: 41.1% HbA: 56.0% HbF: 0.4% HbA2: 2.5% HbS: 39.2% HbA: 58.7% HbA2: 2.1% HbS: 38.6%HbA: 55.8% HbF: 0.8% HbA2: 4.8%
Goldberg et al. [25]	1985	New Mexico, USA NewMexico, USA	18, M, White American 37, M, White American	At Clines Corners, NM 3 hours after arrival into Santa Fe	Physical activity: no Aviation: no Physical activity: no Aviation: no	2195 m 2134m	*Splenectomy*: yes Postoperative course uncomplicated except for left pleural effusion that resolved spontaneously; received nasal oxygen during postoperative period,which lasted 11 days *Splenectomy*: yes Postoperative course uncomplicated; discharged on 7th day	HbS: 45.8% HbA: 51.4% HbA2: 2.8% HbS: 41.0% HbA: 55.0% HbA2: 3.4%
Shalev et al. [45]	1988	Sierra Mountains, CA, USA	22, M, white Israeli Jew of non-Ashkenazi origin	3rd consecutive day of strenuous activity	Physical activity: yes Aviation: no	3536 m	*Splenectomy*: yes	HbS: 46.5% HbA: 50.2% HbF: 1.4% HbA2: 1.9%
Gitlin et al. [24]	1989	Michigan, USA	27, M, Middle Eastern descent	Middle of night	Physical activity: no Aviation: no	None	*Splenectomy*: no Conservative therapy; patient recovered except for an episode of acute tophaceous gout that occurred 9 days after discharge	Unknown
Narasimhan et al. [54]	1990	Unknown	24, M, unknown	Unknown	Physical activity: no Aviation: no	1524 m	*Splenectomy*: no Conservative therapy	Unknown
Sugarman et al. [48]	1990	Durham, NC, USA	43, M, Black	8 days after being admitted for pulmonary thromboembolism	Unknown	None	*Splenectomy: No*	HbS: 39% HbA: 61%
Novielli et al. [37]	1991	Pennsylvania, USA	38, F, Black	Few hours after cocaine use	Physical activity: no Aviation: no	None	*Splenectomy*: no Conservative therapy (a high concentration of cocaine in spleen may have resulted in acute vasoconstriction leading to further lowering oxygen tension)	Unknown
Genet et al. [23]	1996	Unknown	65, F, North African	Unknown	Physical activity: no Aviation: no	None	*Splenectomy*: yes With a follow-up of 2 years, the patient was doing well (there was no arterial hypoxemia before splenic infarction; the patient suffered from multiple severe thrombotic processes without predisposing factors)	HbS: 40.3% HbA: 57.7% HbA2: 2.2%
Bodo et al. [14]	1997	St. Louis, MO, USA	49, F, African American	During sleep	Physical activity: no Aviation: no	None	*Splenectomy*: no Conservative therapy: yes	HbS: 37%
Franklin et al. [21]	1999	Bridgeport, CA, USA Unknown Vail, CO, USA Utah, USA	21, M, African American 20, M, Mexican 30, F, White 34, M, African American	Within 12 hours of arriving in Bridgeport 2 days after descending from altitude 2nd day on vacationUnknown	Physical activity: yes Aviation: no Physical activity: yes Aviation: no Physical activity: yes Aviation: no Physical activity: yes Aviation: no	2042 m 1524m 3048m 2438m	*Splenectomy*: no Stay in hospital was unremarkable (patient had history of G6PD) *Splenectomy*: no Graduation resolution of symptoms throughout his stay in hospital (has subsequently traveled to altitudes of similar altitudes without sequelae) *Splenectomy*: no Conservative therapy and resolution of symptoms; avoided skiing for 2 years but on 4th ski trip at altitude of ~12 000 ft, had a recurrence of symptoms. She returned to sea level with gradual resolution of symptoms *Splenectomy*: yes Postoperative coursewas complicated by left subdiaphragmatic abscess with colonic fistula formation	HbS: 43.1% HbA: 54.0% HbA2: 2.9% HbS: 41.9% HbA: 55.9% HbA2: 2.2% HbS: 39.0% HbA: 61.0% HbS: 37.5% HbA: 58.8% HbA2: 3.7%
Ozgen et al. [41]	1999	Unknown Unknown	26, M, Cyprus 19, M, Cyprus	5 days after complaining of diarrhea Unknown	Physical activity: no Aviation: no Physical activity: no Aviation: no	None None	*Splenectomy*: yes *Splenectomy*: unreported	Unknown Unknown
Tiernan [49]	1999	Sierra Nevada, USA Sierra Nevada, USA	26, M, White American 17, M, White American	Upon ascent to high altitude; chest pain in themiddle of night After fishing for an hour	Physical activity: no Aviation: no Physical activity: no Aviation: no	2830 m 2740m	*Splenectomy*: no Conservative therapy; pain worsened over 3–4 days but resolved after 1 week *Splenectomy*: no Pain resolved in a couple of hours after leaving elevation and was entirely asymptomatic	HbS: 44.4% HbA: 52.4% HbA2: 3.2% HbS: 42.7% HbA: 55.1% HbA2: 2.2%
Symeonidis et al. [55]	2001	Greece	17, M, unknown	24 hours after fever	Physical activity: no Aviation: no	None	*Splenectomy*: no Patient’s coursewas benign; pain subsided after 7 days and fever resolved on the 10th day. He was discharged on the 16th day and follow-up after 3 years was uneventful (the congestion induced by EBV infection and high-grade fevermay have contributed to splenic sequestration and subsequent infarcts)	HbS: 42.0% HbA: 56.0% HbA2: 2.0%
Sheikha [46]	2005	Abha, Saudi Arabia Abha, Saudi Arabia Abha, Saudi Arabia Abha, Saudi Arabia	35, M, Yemeni 32, M, Saudi 23, M, Eritrean 26, M, Southern India	2nd day after arrival to Abha 1st day after arrival to Abha After arrival into Abha After arrival into Abha after visit in lowlands	Physical activity: no Aviation: no Physical activity: no Aviation: no Physical activity: no Aviation: no Physical activity: no Aviation: no	3050 m 3050 m 3050 m 3050 m	*Splenectomy*: yes *Splenectomy*: yes *Splenectomy*: yes *Splenectomy*: yes	HbS: 42.0% HbA: 55.0% HbA2: 3.0% HbS: 40.0% HbA: 57.0% HbA2: 3.0% HbS: 44.0% HbA: 53.0% HbA2: 3.0% HbS: 41.0% HbA: 57.0% HbA2: 2.0%
Malik et al. [32]	2006	Canada	41, M, East Indian	Unknown	Physical activity: no Aviation: no	None	*Splenectomy: no Conservative therapy: yes; analgesia and fluid rehydration*	HbS: 40%
Chamberland [17]	2007	Utah, USA	51, M, African American	Sudden	Physical activity: no Aviation: no	4500 m	*Splenectomy*: no Conservative therapy; was discharged after received supplemental oxygen (had a history of heroin use; he also did not travel to 4500 m because he lived there his entire life)	Unknown
Arora et al. [12]	2008	India India	36, M, Indian 30, M, Indian	Unknown Unknown	Physical activity unknown: Aviation: unknown Physical activity: unknown Aviation: unknown	1676–3962 m 1676–3962m	*Splenectomy*: yes *Splenectomy*: yes	Unknown Unknown
Cook [18]	2008	Cusco, Peru	23, M, European	On ascent	Physical activity: yes Aviation: no	3300 m	*Splenectomy*: yes	HbS: 37.9%
Morishima et al. [33]	2008	Mt. Fuji, Japan	41, F, African American	On ascent	Physical activity: yes Aviation: no	~3776 m	*Splenectomy*: no Conservative therapy; recovered without sequelae (patient had a history of alcoholism and cholelithiasis)	Unknown
Pothula et al. [43]	2008	Mt. Fuji, Japan Mt. Fuji, Japan Mt. Fuji, Japan Mt. Fuji, Japan Mt. Fuji, Japan Mt. Fuji, Japan	23, M, French and African American 26, M, Hispanic (white American) 20, M, African American 24, M, Mediterranean descent 26, M, African American 34, M, African American	During ascent 1 week after climb During ascent (3 hours after began climb) During ascent 1 day after climb During ascent	Physical activity:yes Aviation: no Physical activity: yes Aviation: no Physical activity: yes Aviation: no Physical activity: yes Aviation: no Physical activity: yes Aviation: no Physical activity: yes Aviation: no	2286 m 3755 m 3000 m 2194 m 3775 m 3657 m	*Splenectomy*: no Conservative therapy; symptoms resolved and patient went back to work *Splenectomy*, no Conservative therapy: yes *Splenectomy*, no Conservative therapy; 1 month later, asymptomatic and CT showed improved areas of infarcted spleen *Splenectomy*, yes Discharged 6 days after began hospital stay; returned ~2 weeks later with recurrent left upper quadrant pain *Splenectomy*, yes Returned to fu 11 duty a few weeks after postoperative ly *Splenectomy*, yes Postoperative course was uncomplicated	Unknown Unknown Unknown Unknown Unknown Unknown
Funakoshi et al. [22]	2010	Mt. Fuji, Japan	38, M, Mestizo	During ascent	Physical activity:yes Aviation: no	3400 m	*Splenectomy:* no Conservative therapy: yes; 5-month follow-up was uncomplicated	HbS: 40.5%
Norii et al. [36]	2011	Mt. Fuji, Japan Mt. Fuji, Japan	21, M, African American 41, F, African American	During ascent During ascent	Physical activity:yes Aviation: yes (day after admission to hospital but no increased pain) Physical activity: yes Aviation: yes (day after admission to hospital but no increased pain)	Mt. Fuji: 3776 m Cabin pressure altitude: 2438 m Mt. Fuji: 3776 m Cabin pressure altitude: 2438 m	*Splenectomy: no* Conservative therapy: yes; patient recovered without sequelae *Splenectomy*: no Conservative therapy: yes; patient recovered without sequelae (patient had a previous history of alcoholism)	Unknown Unknown
Abeysekera et al. [10]	2012	Sri Lanka	31, M, Sri Lankan	Peak of Sri Pada (Adam’s Peak)	Physical activity:yes Aviation: no	2243 m	*Splenectomy:* no Conservative therapy: yes; completely recovered (this was his 4th trip to the same mountain during the last 10 years*)*	HbS: 42.6% HbA: 49.3% HbF: 0.9% HbA2: 3.1%
Gotliebetal. [61]	2012	Unknown Unknown Unknown Unknown	45, M, unknown 52, M, unknown 38, M, unknown 45, M, unknown	After 5 hour flight Unknown Unknown Unknown	Physical activity: no Aviation: no Physical activity: no Aviation: no Physical activity: no Aviation: no Physical activity: no Aviation: no	Unknown Unknown Unknown Unknown	*Splenectomy:* no Conservative therapy: yes; after aggressive hydration, pain resolved and patient discharged *Splenectomy*, no Conservative therapy: yes; patient was treated with Coumadin (history of renal cell carcinoma) *Splenectomy*, yes (History of alcohol abuse and chronic pancreatitis) *Splenectomy,* no (History of acute pancreatitis)	HbS: 38.7% Unknown Unknown Unknown
Asfaw et al. [13]	2013	Cleveland, OH, USA	50, F, unknown	Unknown	Physical activity: no Aviation: no	Unknown	*Splenectomy*: no Required endotracheal intubation and initiation of vasopressor support on 3rd day of hospital stay; developed multisystem organ failure after omentectomy, subtotal colectomy, and small bowel resection. Supportive care withdrawn and died (had history of cocaine use and pathology showed vascular congestion with sickled RBC)	Unknown
Gupta et al. [26]	2013	Nanda Devi, Garhwal, Himalayas	21, M, Indian	During ascent	Physical activity: yes Aviation: no	3500 m	*Splenectomy*: no Conservative therapy; patient recoveredwith sequelae	HbS: 38.7% HbA: 58.0%
Murano et al. [34]	2013	San Diego, CA, USA, to Newark, NJ, USA	49, M, African American	After alcoholic beverage in flight	Physically active: no Aviation: yes	Unknown	*Splenectomy*: yes Patient had an uneventful recovery and was discharged	HbS: 43.5%
Scordino et al. [44]	2013	Cusco, Peru	24, M, African American	During hike	Physical activity: yes Aviation: no	Unknown	*Splenectomy*: no Conservative therapy; after returning to US, pain improved but was not resolved. He had follow-upwithin 1 week and did not require surgical follow-up	Unknown
Habibzadeh et al. [57]	2015	Ardabil, Iran	18, M, unknown	After mountain climbing	Physical activity: yes Aviation: no	Unknown	*Splenectomy*: no Conservative therapy: yes; pain was controlledwith opioid analgesics.	HbS: unknown HbA1: 54.1% HbA2: 2.7% HbF: 43.2%
Hota et al. [58]	2015	India India India India India	27, M, unknown 33, M, unknown 24, M, unknown 29, M, unknown 31, M, unknown	Within 12 hours of exposure to altitude Within 24 hours Within 72 hours Within 12 hoursWithin 48 hours	Physical activity: no Aviation: yes Physical activity: no Aviation: yes Physical activity: no Aviation: yes Physical activity: no Aviation: yes Physical activity: no Aviation: yes	3962 m 3962m 3962m 3962m 3962m	*Splenectomy*: yes *Splenectomy*: no Conservative therapy: yes *Splenectomy*: yes *Splenectomy*: no Conservative therapy: yes *Splenectomy*: no Conservative therapy: yes	Unknown Unknown Unknown Unknown Unknown
Nofal et al. [35]	2015	Unknown	7, M, African American	During acute phase of EBV infection	Physical activity: no Aviation: no	None	*Splenectomy*: no Conservative therapy: yes; with RBC transfusion, hydration, and pain control. Patient was discharged home once stable	HbS: 33% HbA: 63.9% HbA2: 3.1%
Seegars [8]	2015	Columbia, SC, USA	18, F, African American	Spontaneous	Physical activity: no Aviation: no	Low (91 m)	*Splenectomy*: no Conservative therapy: yes; 4 days after discharged, returnedwith fever and increasing pain in left upper abdomen. She was subsequently dischargedwith 48 hours	HbS: 39.2% HbA: 58.6% HbA2: 2.3%
Hayashi et al. [27]	2016	Japan	20s, M, African American	While climbing mountain	Physical activity: yes Aviation: no	>3000 m	*Splenectomy*: no Conservative therapy: yes; led to improved symptoms	Unknown
Walcott-Sapp et al. [50]	2016	Oregon, USA	21, M, Spanish Italian-Irish-Seminole Tribe descent	1 hour within arrival	Physical activity: no Aviation: no	2712 m	*Splenectomy*: no Conservative therapy; diet was slowly advanced and pain was controlled	HbS: 40.1% HbA: 56.8% HbA2: 3.1%
Magro et al. [31]	2017	Italy	11, M, Nigerian	Two days after flying home	Physical activity: no Aviation: yes	Unknown	*Splenectomy*: no Conservative therapy	HbS: 40.6% HbA: 55.2% HbA2: 3.5% HbF: 0.7%
O’Shea et al. [39]	2017	Ethiopia	24, M, Sudanese	Upon landing in Ethiopia	Physical activity: no Aviation: yes	Unknown	*Splenectomy*: no Conservative therapy: yes; symptoms improved over 6 days	HbS: 39%
Patro et al. [42]	2017	Bangalore, India	44, M, Indian	Upon ascent	Physical activity: unknown Aviation: no	3350 m	*Splenectomy*: yes	HbS: 42.55% HbA: 53.87% HbA2: 3.57%
Sinha et al. [47]	2017	India India	55, M, Indian 27, M, Indian	At the end of journey At the end of journey	Physical activity: yes Aviation: yes Physical activity: yes Aviation: yes	3888 m 3888 m	*Splenectomy*: no Conservative therapy: yes; symptoms subside in 10 days *Splenectomy*: no Conservative therapy: yes; symptoms subside in 5 days	HbS: 29.8% HbS: 32%
Alabbadi et al. [11]	2018	Saudi Arabia	24, M, Saudi Arabian	During flight	Physical activity: yes Aviation: yes	None	*Splenectomy*: no Conservative therapy: yes;: pain control	HbS: 40% HbA1: 54.6% HbA2: 1.8% HbF: 3.6%
Fernando et al. [56]	2018	Hambantota, Sri Lanka	26, M, unknown	During descent	Physical activity: yes Aviation: yes	2243 m	*Splenectomy*: no Conservative therapy: yes; discharged on oral penicillin and immunization; platelets rose gradually	HbS: 38.6% HbA: 50.6%
Yanamandra et al. [51]	2018	India	24, M, Indian	Upon ascent	Physical activity: yes Aviation: no	3500 m	*Splenectomy*: no Conservative therapy: yes; recurrent symptoms over next year or so	Unknown
Gross et al. [52]	2018	Unknown	19, M, African American	Unknown	Physical activity: unknown Aviation: Unknown	Unknown	*Splenectomy: no Conservative therapy: unknown*	HbS: 39.7%
Alsinan et al. [62]	2019	Unknown	15, M, unknown	Unknown	Physical activity: unknown Aviation: unkown	Unknown	*Splenectomy: yes Conservative therapy: unknown*	HbS: 45%
Kamada et al.[59]	2019	Japan	38, M, unknown	While climbing Mt. Fuji	Physical activity: yes Aviation: no	2500 m	*Splenectomy: no Conservative therapy: unknown*	unknown
Moideen et al. [53]	2019	Tamil Nadu, India	27, M, Southern India	Unknown	Physical activity: unknown Aviation: unknown	unknown	*Splenectomy: no Conservative therapy: yes; fluids*	HbS: 39.3%
Rao E et al. [60]	2019	Denver, CO, USA Denver, CO, USA Denver, CO, USA	17, M, unknown 13, M, unknown Unknown, F, unknown	2 days after traveling into Frisco, CO 2 days after traveling into Frisco, CO 2 days after traveling into Frisco, CO	Physical activity: unknown Aviation: yes Physical activity: unknown Aviation: yes Physical activity: unknown Aviation: yes	~2800 m ~2800 m ~2800 m	*Splenectomy: no Conservative therapy: pain control Splenectomy: no Conservative therapy: unknown Splenectomy: no Conservative therapy: unknown*	Unknown Unknown Unknown
